# Tropical cyclones alter short-term activity patterns of a coastal seabird

**DOI:** 10.1186/s40462-019-0178-0

**Published:** 2019-10-28

**Authors:** Bradley P. Wilkinson, Yvan G. Satgé, Juliet S. Lamb, Patrick G. R. Jodice

**Affiliations:** 1 Department of Forestry and Environmental Conservation, Clemson University, Clemson, SC 29634 USA; 2 South Carolina Cooperative Fish and Wildlife Research Unit, Clemson, SC 29634 USA; 3 Department of Natural Resource Science, University of Rhode Island, Kingston, RI 02881 USA; 4 U.S. Geological Survey South Carolina Cooperative Fish and Wildlife Research Unit, Clemson, Clemson, SC 29634 USA

**Keywords:** Environmental disturbance, EMbC, Hurricane, Seabird, Tracking, Sheltering behavior

## Abstract

**Background:**

Mobile organisms in marine environments are expected to modify their behavior in response to external stressors. Among environmental drivers of animal movement are long-term climatic indices influencing organism distribution and short-term meteorological events anticipated to alter acute movement behavior. However, few studies exist documenting the response of vagile species to meteorological anomalies in coastal and marine systems.

**Methods:**

Here we examined the movements of Eastern brown pelicans (*Pelecanus occidentalis carolinensis*) in the South Atlantic Bight in response to the passage of three separate hurricane events in 2 years. Pelicans (*n* = 32) were tracked with GPS satellite transmitters from four colonies in coastal South Carolina, USA, for the entirety of at least one storm event. An Expectation Maximization binary Clustering algorithm was used to discretize pelican behavioral states, which were pooled into ‘active’ versus ‘inactive’ states. Multinomial logistic regression was used to assess behavioral state probabilities in relation to changes in barometric pressure and wind velocity.

**Results:**

Individual pelicans were more likely to remain inactive during tropical cyclone passage compared to baseline conditions generally, although responses varied by hurricane. When inactive, pelicans tended to seek shelter using local geomorphological features along the coastline such as barrier islands and estuarine systems.

**Conclusions:**

Our telemetry data showed that large subtropical seabirds such as pelicans may mitigate risk associated with spatially-extensive meteorological events by decreasing daily movements. Sheltering may be related to changes in barometric pressure and wind velocity, and represents a strategy common to several other classes of marine vertebrate predators for increasing survival probabilities.

## Background

Mobile organisms display common movement syndromes across vertebrate taxa, with movements based on both intrinsic (e.g. body condition) and extrinsic factors (e.g. resource availability) [1]. While intrinsic variation operates on the level of the individual, extrinsic factors acting concurrently on groups of individuals have a role in determining the movement behavior of populations [2]. Among these extrinsic factors in marine and coastal systems are climatic variations that affect distributions on monthly, yearly, or decadal timescales. Long-term drivers include extensive and cyclic events such as seasonality [3–5], oscillation events (e.g. El Niño Southern Oscillation) [6, 7], and oceanographic-atmospheric regime coupling [8–11]. Extrinsic drivers of animal movement also occur at more local scales, where acute meteorological events such as storms can influence animal movement from hours to weeks [12, 13]. These short-term events are expected to fluctuate stochastically compared to longer-term climatic drivers, and therefore the extent of and mechanisms by which each affect movement may be variable. While the spatial impacts of macroscale events are relatively well-studied, effects of shorter-term acute drivers (e.g. local storms) are less known [14].

While organisms may respond to seasonally-typical meteorological conditions in repeatable and often predictable ways, anomalous conditions offer an opportunity to examine behavioral responses to environmental stressors that occur stochastically [15–17]. Among the most disruptive meteorological events in coastal and marine systems are hurricanes and tropical storms (also called cyclones or typhoons). These spatially-extensive, temporally-focused natural perturbations can affect coastal geomorphology, alter local oceanography, and induce widespread mortality among wildlife populations [18–20]. Typically categorized by relative severity, they are regularly-occurring yet unpredictable phenomena [21]. Hurricanes introduce extreme wind velocities, elevated tidal surges, intense rainfall, widespread flooding, and chaotic sea surface conditions to the local system, and therefore have the potential to reduce organism fitness directly (e.g. mortality events) and indirectly (e.g. reduced foraging opportunities) [22].

Species that occupy ecosystems regularly subjected to hurricanes demonstrate behavioral modifications for increasing survival during cyclonic activity, although direct studies appear limited [14]. For example, marine species commonly display one of two contrasting strategies for mitigating negative effects from intense but short-duration weather events; relocation and sheltering in place. Studies of elasmobranchs (e.g. juvenile blacktip sharks (*Carcharhinus limbatus*)) have demonstrated increased movement rates upon the approach of a cyclone indicating relocation from shallow nursery areas to deeper, offshore water that is less prone to disturbance [23, 24]. Conversely, Florida manatees (*Trichechus manatus latirostris*) remain in the same discrete patch during passage of a cyclonic event, with daily movements contained within areas utilized prior to cyclonic exposure [25]. Littoral abundance of sea kraits (*Laticauda* spp.) in Taiwan appears to be influenced by cyclonic events, with individuals likely seeking shelter among coastal geologic features such as sea caves [26]. Results from loggerhead and hawksbill sea turtles (*Caretta caretta* and *Eretmochelys imbricate*, respectively) indicate marked changes in swimming and diving behavior during storm interaction, although with variable and sometimes contrasting responses depending on breeding stage [27–29].

Of particular utility for examining differential responses to cyclonic events, seabirds present a group of taxonomically and morphologically diverse organisms often impacted by marine storms. For example, smaller-bodied pelagic seabirds may attempt to avoid or circumnavigate an approaching hurricane [14, 30]. Individuals unable to do so may be displaced far from their preferred habitat (often inland), leading to the observed wrecks of these species following major events (e.g. [31]). Conversely, larger-bodied coastal-dwelling species may reduce daily activities and attempt to shelter during storm passage, but this remains unexamined. Variation in hurricane response may also differ by life stage in addition to morphology [14]. Understanding how various seabird species respond to large-scale environmental irregularities may therefore clarify apparent discrepancies in displacement susceptibility [30]. However, due to the stochastic and unpredictable nature of hurricane events, as well as the difficulties and dangers of collecting data on animal movement during these times, published literature is lacking on this topic particularly for larger-bodied coastal-dwelling species.

As part of ongoing research examining movement patterns of Eastern brown pelicans (*Pelecanus occidentalis carolinensis*) in the South Atlantic Bight, we report the behavioral strategies utilized by two cohorts of satellite-tracked individuals in coastal South Carolina and Georgia during the passage of three hurricane events. The Eastern brown pelican is a large-bodied coastal seabird with breeding colonies distributed along barrier and estuarine islands ranging from tropical to temperate waters of the western North Atlantic. As a facultative migrant, the brown pelican displays a range of individual post-breeding movement strategies [32], which when combined with timing of departure and location of breeding colony, annually exposes many individuals to potential cyclonic events throughout their range. During peak hurricane activity in the South Atlantic Bight (late August to September), adult pelicans may variably disperse from the breeding colony but are generally not yet engaged in migratory behavior (B.W. pers. obs.). We hypothesized that the movement behavior of individual pelicans would correlate with meteorological condition during passage of a hurricane by either (a) increasing movement activity and fleeing the storm or (b) decreasing movement activity and sheltering in place.

## Methods

### Study area

**Fig. 1:**
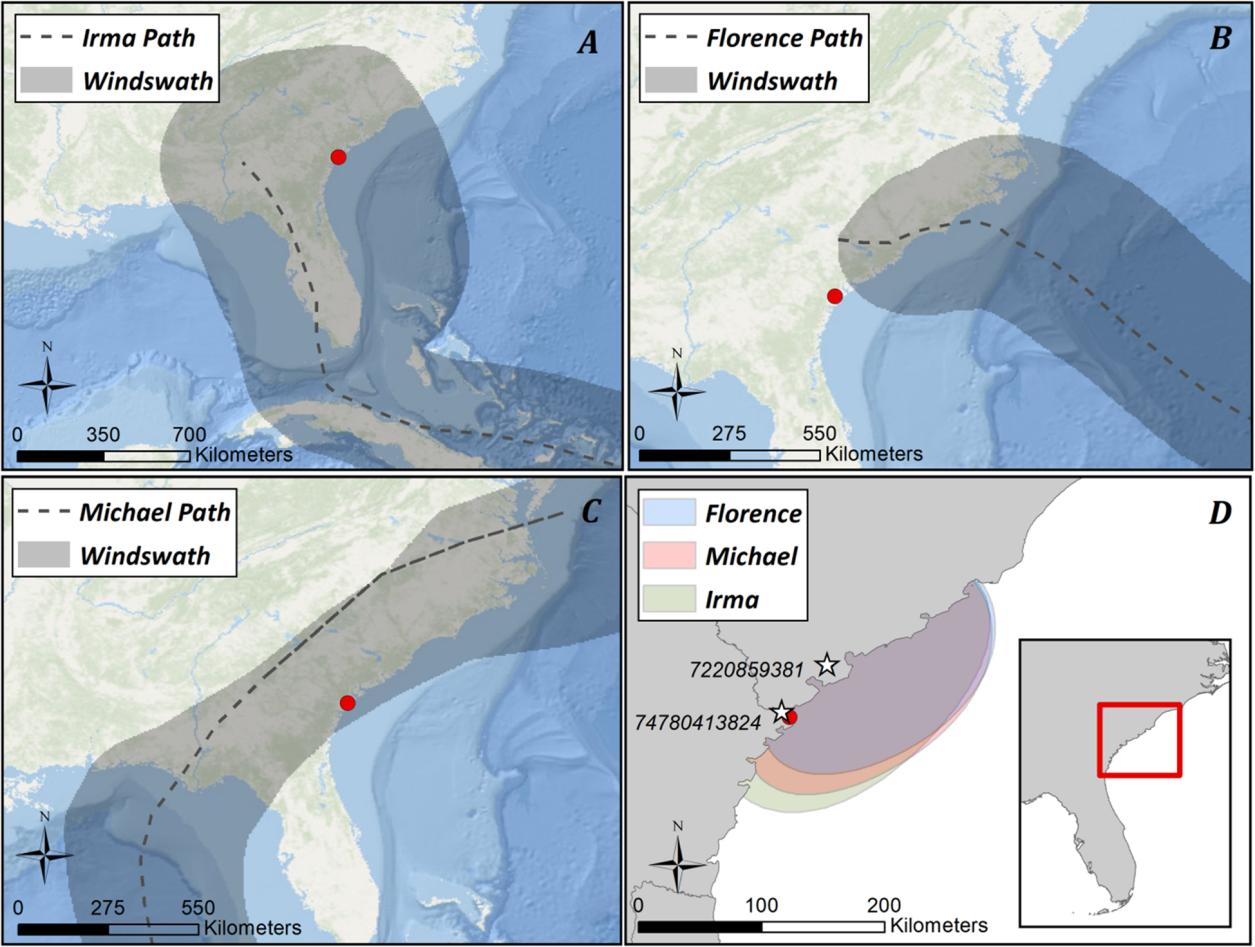
Path and windswath extent of Hurricanes Irma (**a**), Florence (**b**), and Michael (**c**) in the South Atlantic Bight with (**d**) 25% utilization distribution obtained from kernel density analysis of tracked pelicans during the month corresponding to the passage of each hurricane. White stars represent locations of meteorological data collection, with NOAA station identification numbers. Red dots represent Savannah, Georgia, USA. Hurricane data obtained from the NOAA National Hurricane Center and Central Pacific Hurricane Center (https://www.nhc.noaa.gov/gis/)

We conducted our study in the South Atlantic Bight, USA, which extends from the Cape Fear River Basin to approximately Cape Canaveral (Fig. 1). The coast here is characterized by a complex geomorphology of barrier islands, estuaries, and salt marshes. The area supports ca. 15 brown pelican colonies annually (active breeding from April – September) and many of the beaches and islands are used as migratory stopover, staging, or wintering grounds for this species and others [33].

### Satellite transmitter deployments

Nesting pelicans were outfitted with GPS satellite transmitters (GeoTrak Inc., North Carolina, USA) at four colonies in coastal South Carolina (Bird Key Stono, 32° 38′ N, 79° 58′ W, *n* = 21; Castle Pinckney, 32° 46′ N, 79° 54′ W, *n* = 12; Marsh Island, 32° 59′ N, 79° 33′ W, *n* = 7; Deveaux Bank, 32° 32′ N, 80° 10′ W, *n* = 5). Colony size ranged from ca. 50–2000 pairs. Deployments commenced during the chick-rearing stage (May–July) of the 2017 and 2018 breeding seasons. Transmitters weighed ~ 65 g (10 × 3.5 × 3 cm) and constituted ≤3% body mass of instrumented individuals (range = 2475–4350 g), the recommended threshold for large seabirds [34]. Briefly, nest-attending adults were captured via either neck or leg noose and equipped with a solar GPS Platform Terminal Transmitter dorsally using a backpack-style harness system. For a description of specific attachment procedures, see [35]. During the post-breeding stage of deployment (September – November), units were programmed to record 10 locations per day at 90 min intervals between the hours of 01:00–23:30 GMT and were duty-cycled on an 8 h on to 36 h off activity schedule. Unit error was assumed to be similar to that of [32], i.e. 4.03 ± 2.79 m.

### Hurricane events

Our opportunistic analysis of pelican movement in relation to hurricane activity includes three storm events. On 10 September 2017, Hurricane Irma made landfall in southwestern Florida, USA, as a Category 4 tropical cyclone. Over the subsequent 1.5 days, Irma proceeded north along the coast of western Florida before weakening and degenerating near the central Georgia-Alabama border. Although the storm was centered mainly along the Gulf coast of Florida, much of the southeastern Atlantic seaboard was affected by the outer cyclonic bands (Fig. 1).

Hurricane Florence made landfall on 14 September 2018 in southern North Carolina, USA, as a reduced Category 1 tropical cyclone, having been a Category 4 cyclone 4 days prior. Florence tracked inland in a southeasterly direction as it weakened, degenerating over West Virginia, USA, three days after landfall, affecting predominantly the coastal Carolinas (Fig. 1).

Less than 1 month later, Hurricane Michael made landfall in the panhandle of Florida on 10 October 2018 as a Category 4 tropical cyclone. Michael followed a northeasterly trajectory after landfall, weakening incrementally over the southeastern United States before restructuring as an extratropical cyclone 2 days later off the Mid-Atlantic coast (Fig. 1). Similar to Irma, Michael impacted much of the Atlantic seaboard due to the trajectory, strength, and spatial extent of the storm.

### Meteorological data

A kernel density analysis was used to identify the core spatial area utilized by instrumented pelicans during each hurricane event. Subsequent utilization distributions (UDs) were used to determine a representative location for assessing pelican response to meteorological indices. This approach allowed for the acquisition of meteorological data that would represent shared conditions for the greatest number of individuals throughout the tracking period. We used only locations recorded during the calendar month of the respective hurricane event, which corresponded with peak cyclonic activity but limited seasonal changes in weather. Distributions therefore reflected core use areas during the entire passage of the cyclone as well as the remainder of the month in which the cyclone occurred. Erroneous locations were identified and removed through a combination of visual inspection (e.g. consecutive locations separated by unrealistic distances) and a speed filter of ≥65 km per hour [36]. Kernel bandwidth was determined using R statistical software (v 3.4.2.) through a plug-in bandwidth selector in package *ks* [37]. Locations within the 25% UD (i.e. core range) identified in the kernel density output during the month of each respective hurricane (grid = 400, extent = 0.4°) were then used to assess movement patterns in relation to storm events. Roughly, the area of highest use by pelicans during these time periods paralleled the coastline from central South Carolina to north-central Georgia (Fig. 1). Individual pelicans located outside of the prior 25% UD at the time of hurricane passage (e.g. in Chesapeake Bay) were manually excluded from further analysis, as well as individuals for whom movement data was not complete for the entire time period.

Meteorological data were obtained via the National Oceanic and Atmospheric Administration (NOAA) National Centers for Environmental Information from the Hunter U.S. Army Airfield, Savannah, Georgia (station 74780413824), to represent conditions experienced during Hurricane Irma, and from the Marine Corps Air Station Beaufort, Beaufort, South Carolina (station 72208593831), to represent conditions during Hurricanes Florence and Michael (https://www.ncdc.noaa.gov/). These sites were within the 25% UD in the kernel density analysis. Although spatially similar, multiple weather locations were required as neither station had complete data for all three hurricane events in totality. Meteorological data were collected hourly and spanned the entire month of each cyclonic event. Data were requested 04 November 2017, 28 November 2018, and 12 December 2018, respectively.

### Behavioral clustering

**Fig. 2:**
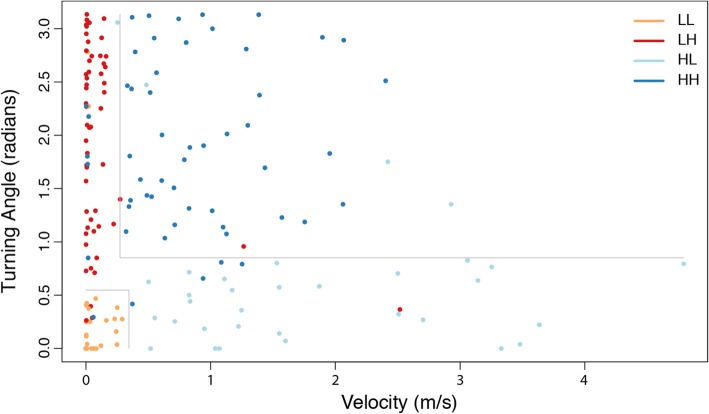
Example scatterplot of Expectation Maximization binary Clustering (EMbC) discretization for one individual Eastern brown pelican in the South Atlantic Bight from 6 to 24 September 2017. Gray lines represent delimiters for categorizing the four possible behavioral states. Note that delimiters do not determine a perfect partition of the variable space, and therefore do not converge perfectly on a graphical plane. Additionally, some points are within the delimiters of separate behavioral states; this is a result of the applied smoothing parameter. See [38] for additional details. All points labeled LH, HL, and HH represent active states; LL represents an inactive state

We used an Expectation Maximization binary Clustering (EMbC) algorithm to derive biologically-relevant behavioral states for individual brown pelicans [38]. EMbC uses unsupervised relationships between successive locations incorporating path distance and tortuosity (i.e. velocity and turning angle) to infer underlying behavioral processes. EMbC is particularly appropriate for remotely-sensed location data as it accounts for spatial and temporal correlations and uncertainties in the input features and is robust to spatial data collected at relatively long intervals [39]. Critically, EMbC is capable of producing biologically-relevant classifications for locational data recorded at timescales relevant to the current study (e.g. [40]). Each point within individual tracks was clustered into one of four categories: low velocity/ low turning angle (LL), low velocity/ high turning angle (LH), high velocity/ low turning angle (HL), and high velocity/ high turning angle (HH) (Fig. 2). These four behavioral nodes were biologically interpreted as corresponding to inactive, localized search, commuting, and dispersive search behaviors, respectively. Following [38], a post-processing smoothing procedure was applied based on consecutive behavioral correlations to manage temporally-irregular data. This smoothing procedure searches for clusters of the same behavioral assignment that contain a single point of a different classification, and adds additional likelihood weight to that single point belonging to the larger cluster, a feature explicitly implemented in state-space models. In this way, the smoothing procedure favors homogenized bouts of behavior instead of single-point behavioral switches during clusters of equal assignment. We also calculated mean step length (distance between successive points) and net displacement (maximum distance from the first location in the series) for descriptive purposes. Each point was finally matched temporally to the closest hourly meteorological variable for statistical analysis.

### Statistical analyses

We assessed the effects of meteorological drivers on pelican behavioral state with multinomial logistic regression following [41]. To simplify model interpretation and to examine activity patterns more accurately matched to the temporal resolution of the data, models were conducted on a reduced set of two behavioral nodes classified as either active (including localized search, commuting, and dispersive search; LH, HL, and HH, respectively) or inactive (LL). Environmental variables of interest (barometric pressure and wind velocity) were chosen a priori based on data completeness, relevance to cyclonic activity, and probability of being sensed by individual pelicans [14].

Both tracking and meteorological data were further subset to exclude other potentially confounding anomalous conditions. We defined an anomalous event as a barometric pressure reading ≥1 SD from the monthly mean. Only data collected from the end of the last pressure anomaly pre-cyclone to the first pressure anomaly post-cyclone were therefore included in our regression analysis, thus creating a temporal segment of activity that was exclusively characterized by ‘baseline’ conditions with the exception of the cyclonic event. Significant differences of barometric pressure and wind velocity between study periods were assessed via Kruskal-Wallis chi-squared tests, with Wilcoxon rank sum tests used when significant differences were found.

Four multinomial logistic regression models were fit to the data using R package *mlogit* [42], including a null model, single-effect wind velocity model, single-effect barometric pressure model, and global model including both wind velocity and barometric pressure. Model selection was performed within each set using Akaike’s Information Criterion (AIC), with the best-performing model indicated by the lowest AIC value. Given low AIC similarity between models, we did not model average. Environmental variables were interpreted as having a significant effect on individual behavioral states at *p* < 0.05. We further assessed transition probabilities using the top-performing model, with the null state (i.e., reference level) defined as inactive (i.e., the probabilities are reflective of transitioning from inactivity to activity).

## Results

After removal of individuals with incomplete tracks and those located outside of the 25% UD, 32 instrumented Eastern brown pelicans remained in the sample population for Hurricanes Irma (*n* = 18), Florence (*n* = 16), and Michael (*n* = 12). Due to the multi-year duration of tag deployment as well as the temporal spacing of cyclonic events, some individuals were tracked for more than one event (2 events, *n* = 8; 3 events, *n* = 3).

**Fig. 3:**
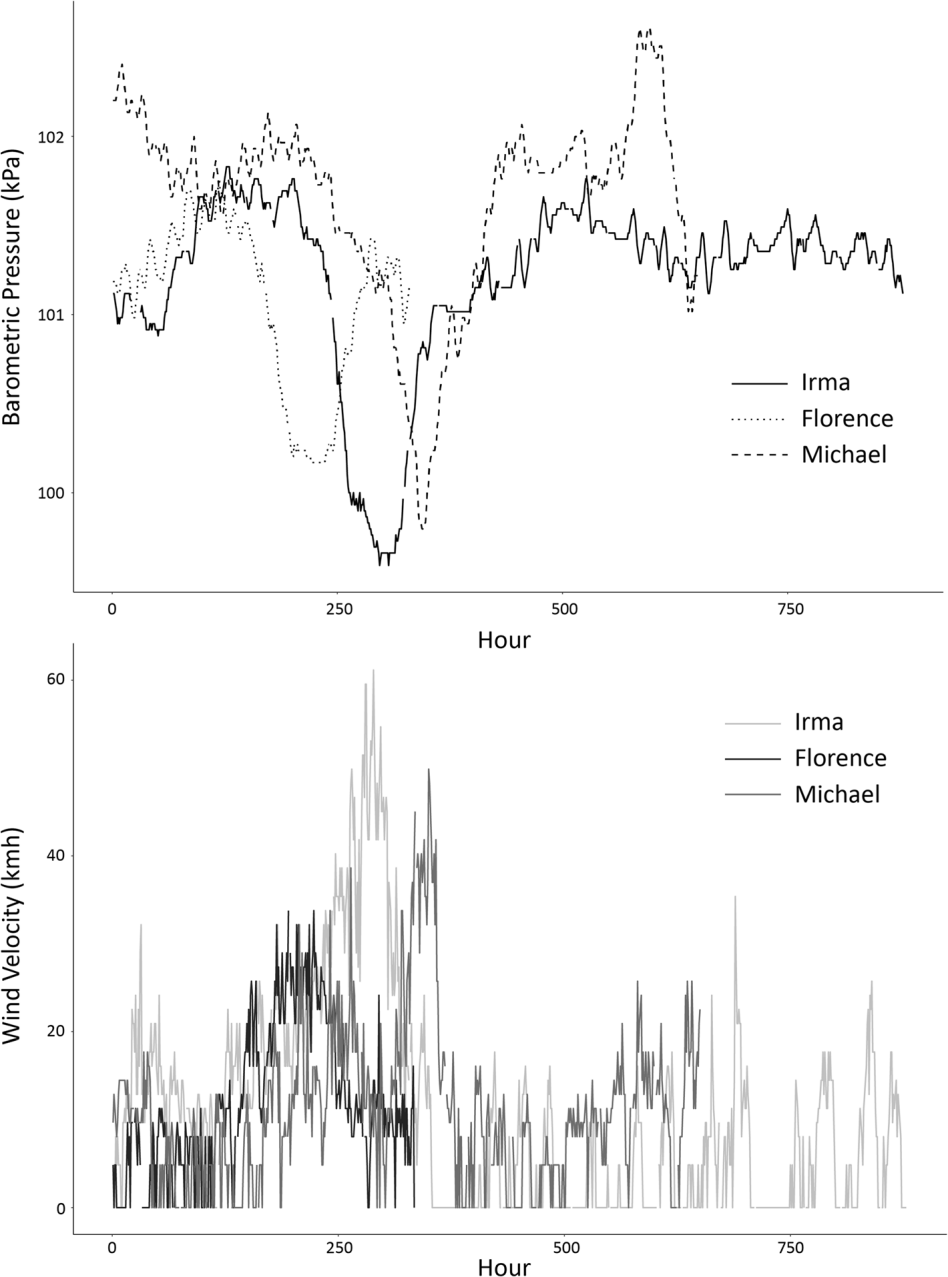
Hourly barometric pressure and wind velocity recorded during the study period of Hurricanes Irma, Florence, and Michael. Solid or light grey lines represent Irma, dotted or black lines represent Florence, and dashed or medium grey lines represent Michael, respectively

Hourly barometric pressure and wind velocity were relatively consistent throughout each defined study period with the exception of hurricane passage (Fig. 3). Local minima of barometric pressure and local maxima of wind velocity were both greater than one standard deviation away from the monthly mean during the day that the center of the storm passed through the study area (Table 1), indicating anomalous conditions.

**Table 1:** Summary of meteorological data for each cyclonic event in the South Atlantic Bight from 2017 to 2018 with the number of pelican locations recorded during each study period (temporal range of ‘baseline’ conditions, defined in-text)

Cyclone	BP Monthly Mean (kPa)	BP Minimum (kPa)	WV Monthly Mean (km/h)	WV Maximum (km/h)	Study Period	Locations (*n*)
Irma	101.25 ± 0.43^a^	99.59^a^	10.4 ± 10.9^a^	61.2^a^	6–24 Sept. 2017	2901
Florence	101.56 ± 0.49^b^	100.17^b^	10.2 ± 7.2^b^	33.8^b^	9–18 Sept. 2018	1323
Michael	101.63 ± 0.58^c^	99.80^c^	11.3 ± 8.4^b^	49.9^b^	1–20 Oct. 2018	2124

*BP* = barometric pressure, *WV* = wind velocity. Letters within each column indicate significant differences between study periods based on Wilcoxon rank sum test

Barometric pressures were significantly different during each period of study (Kruskal-Wallis χ^2^ = 442.27, *p* < 0.001), with lower values during Hurricane Irma than Hurricanes Florence (Wilcoxon rank sum test *Z* = − 5.26, *p* < 0.001) and Michael (*Z* = − 18.66, *p* < 0.001), and significantly lower values during Hurricane Florence than Hurricane Michael (*Z* = − 16.19, *p* < 0.001). Significant differences likewise existed between measured wind velocities (Kruskal-Wallis χ^2^ = 15.89, *p* < 0.001), but not between every event. Wind velocities were higher during Hurricane Irma than Hurricanes Florence (Wilcoxon rank sum test *Z* = − 3.39, *p* < 0.001) and Michael (*Z* = − 2.39, *p* = 0.017), but wind velocities between Hurricane Florence and Hurricane Michael were not significantly different (*Z* = − 1.66, *p* = 0.096).

Pelicans tended to make relatively short daily movements during each period of analysis, and these movements were typically ≤5 km seaward from the immediate coastline ($\underset{\_}{x}$ step length = 3.8 ± 7.1 km, range = 0–94.9 km). Individual pelicans displayed both sedentary and dispersive behavior at the regional level, consistent with individual variation in post-breeding dispersal ($\underset{\_}{x}$ net displacement = 51.7 ± 69.0 km, range = 0–267.4 km). Behavioral assignments discretized by the EMbC algorithm were more likely to be in active state (66.1 ± 17.9%) than in inactive state (33.4 ± 17.8%). Multinomial logistic regression and AIC-driven model selection indicated global models (i.e., barometric pressure + wind speed) as best candidates for explaining pelican behavioral state probabilities during both Hurricanes Irma and Florence (ΔAIC_c_ = 11.52 and 9.38, respectively). Both the global model and a model including only wind speed were selected as best candidates during Hurricane Michael (ΔAIC_c_ = 1.51).

During Hurricane Irma, individuals were significantly more likely to transition from an inactive state to an active state when barometric pressure increased, but were significantly more likely to remain in an inactive state when wind velocity increased (Table 2). The odds of an individual transitioning from an inactive state to an active state decreased by 0.91 for every unit decrease in barometric pressure while the odds of an individual transitioning from an inactive state to an active state decreased by 0.84 for every unit increase in wind velocity. During Hurricane Florence, individuals were significantly more likely to transition from an inactive state to an active state given an increase in barometric pressure as well as an increase in wind velocity (Table 2). The odds of an individual transitioning from an inactive state to an active state decreased by 0.77 for every unit decrease in barometric pressure and increased by 1.20 for every unit increase in wind velocity. According to the global model, during Hurricane Michael individuals were significantly more likely to remain in an inactive state given an increase in wind velocity (Table 2). There was no significant relationship between barometric pressure and activity. The intercept was the only significant coefficient in the model that included only wind speed, and is therefore not reported. The odds of an individual transitioning from an inactive state to an active decreased by 0.90 for every unit increase in wind velocity.

**Table 2:** Results of pooled behavioral state modeling using multinomial logistic regression in relation to environmental variables representing passage of Hurricanes Irma (I), Florence (F), and Michael (M)

Variable	Coefficient	Standard Error	t-value
Intercept (I)**	0.9991	0.0424	23.5649
Barometric Pressure (I)*	0.0978	0.0462	2.1166
Wind Speed (I)**	−0.1744	0.0474	−3.6760
Intercept (F)**	0.6370	0.0584	10.9010
Barometric Pressure (F)**	0.2600	0.0776	3.3498
Wind Speed (F)*	0.1822	0.0780	2.3360
Intercept (M)**	0.3678	0.0443	8.2979
Barometric Pressure (M)	−0.0872	0.0466	−1.8701
Wind Speed (M)*	−0.1088	0.0467	−2.3314

Asterisks represent *p*-values for significant terms (*p* < 0.05 = *, *p* < 0.001 = **)

## Discussion

Based on results from EMbC analysis and multinomial logistic regression, we demonstrate that Eastern brown pelicans in the South Atlantic Bight respond to the passage of spatially-extensive cyclonic events by increasing time of inactivity, regardless of initial landfall proximity. We also found that barometric pressure and wind velocity were significant predictors of behavioral state, indicating that individuals may adjust their behavior in response to meteorological changes associated with storm conditions.

Among several classes of marine taxa, perturbations in barometric pressure appear to be a consistent predictor of behavioral change during storm events [23, 24, 26, 43]. Evidence from terrestrial ecosystems also indicate that some bird species adjust their behavior in response to sudden decreases in atmospheric pressure. For example, [44] demonstrated that declining barometric pressure instigated an increase in food intake for captive white-crowned sparrows (*Zonotrichia leucophrys*). Similar results were obtained by [45] in white-throated sparrows (*Zonotrichia albicollis*). Our data suggest that pelicans likewise modify their behavior given sudden decreases in barometric pressure. Although fine scale fluctuations in absolute pressure may not be meaningful, or possibly even detectable, precipitous declines like those experienced during cyclonic events could indicate environmental conditions detrimental to individual condition.

Our results also show a strong predictive relationship between wind velocity and behavioral state in brown pelicans. Although wind velocity is infrequently considered as a driver of behavioral changes in strictly aquatic species compared to barometric pressure, it is reasonable to conclude that avian species requiring flight to forage or relocate would be especially sensitive to anomalous wind conditions. Observations of the movements of red-footed boobies (*Sula sula)* and great frigatebirds (*Fregata minor*) during cyclonic activity in the Southern Hemisphere suggest that individuals of these species are able to detect approaching gale-force winds as an indicator of an impending cyclone and utilize them for avoidance behavior, although this relationship was not explored quantitatively [14]. In contrast to more pelagic species, pelican locomotion may be hampered by severely elevated wind velocities [46, 47], precluding avoidance behavior. Intrinsic differences in wing morphology (i.e. aspect ratio) and flight characteristics support this differential response in flight to increasing wind conditions [48], although life stage and breeding status may be relevant as well [14].

Model results suggest that behavioral responses to storm activity may also vary with the magnitude of the storm itself. Of the three cyclonic events we assessed, meteorological conditions during Hurricane Irma included the highest and lowest absolute values for wind velocity and barometric pressure, respectively, and were significantly different from both Florence and Michael. These anomalous conditions were also maintained over a longer duration of time compared to other events. Our models for pelican behavior during Hurricane Irma indicated that both low barometric pressure and high wind velocity were highly significant predictors of inactivity; however, this trend differed among cyclonic events (Table 2). For example, pelicans experienced significantly lower wind velocities during Hurricane Florence and for a shorter duration. As such, our models showed a positive relationship between wind speed and activity, but this may be an artefact of the overall lower magnitude of wind velocity change from baseline during the event period. Similarly, Hurricane Michael was characterized by a moderate but relatively sudden decrease in barometric pressure, and models indicated an unexpected negative relationship with pelican activity (Fig. 3). It should be noted, however, that this term was non-significant in the top model and that a model including only wind velocity was also highly supported. We posit that cyclone characteristics contribute significantly to the degree of behavioral modification among individuals, and that events with a higher magnitude of change from ‘baseline’ over a longer period of time, such as experienced during Hurricane Irma, result in a greater reduction of activity than comparably weaker events. Events of greater magnitude may be more easily sensed by pelicans and with greater certainty of producing inclement conditions, eliciting a more detectable behavioral response.

Alternative sources of variation in model coefficients include sample size discrepancies, manifested as ‘pelican-hours’ (i.e., the number of tracked pelicans multiplied with the number of hours of each study period). For example, fewer individual pelicans were tracked during Hurricane Florence (*n* = 15) in comparison to Hurricane Irma (*n* = 18), exacerbated by a 10-day study period compared to a 19-day study period, respectively. This resulted in over twice as many ‘pelican-hours’ and subsequent behavioral classification points for Hurricane Irma than Florence, potentially adding greater resolution to behavioral contrasts between hurricane and non-hurricane time series. Models may also be sensitive to the magnitude of behavioral change displayed during different events, with comparatively weak reductions in activity being undetected. Additional data would therefore be required to determine if spatial sampling rate during data collection or storm characteristics (e.g. duration and intensity) would have greater influence on the magnitude of behavioral change detectable during future cyclonic events.

Timing of cyclones with respect to date and stage of the breeding cycle may also affect the overall activity rates. While Hurricanes Irma and Florence both occurred in early-to-mid September (soon after the end of chick-rearing), Hurricane Michael made landfall in early October, nearly a full month later in the annual cycle. Pelicans may endogenously be less active during later months as temperatures drop and energy maintenance becomes more prominent, but this requires further study, as does the extent of post-fledging care in this species.

**Fig. 4:**
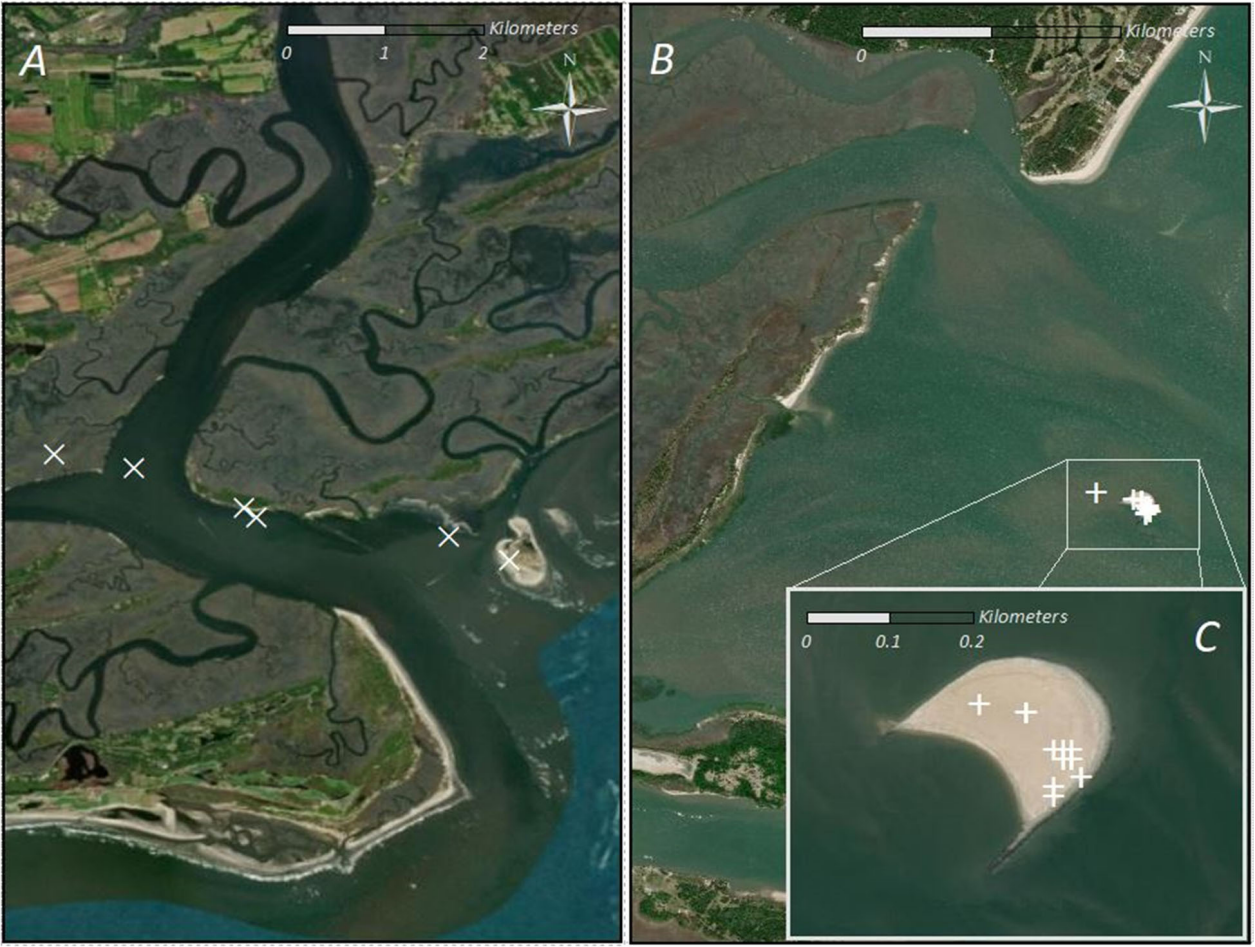
Example habitats used by Eastern brown pelicans during the passage of Hurricane Irma through the South Atlantic Bight on 11 September 2017. **a** Individual pelican moving progressively inland up a coastal river, settling on both a barrier island and in an estuary. **b** Individual pelican sheltering on a small barrier island for the duration of the cyclone, with **c** inset of the island

Access to readily-available refugia in the form of barrier islands and estuarine systems may also positively act upon coastal seabirds to remain stationary during extreme meteorological conditions (Fig. 4). As strictly pelagic seabird species typically remain offshore for resource acquisition, access to shelter during the passage of a hurricane is functionally negligible. It is unclear whether pelagic species would attempt resting on the surface of the water as a sheltering strategy, given the likely turbulent conditions, probable reduction in foraging opportunity, and ability to maintain efficient flight even during severe wind conditions. Indeed, some tropical species appear to make use of terrestrial structures when cyclones approach breeding colonies and access to refugia is available, yet display avoidance behavior when encountering a cyclone at sea [14]. Visual inspection of pelican tracks indicate a frequent use of protected estuarine habitats during severe storms, although further analysis of habitat associations is needed to determine the magnitude and significance of these relationships.

Lastly, the behavioral changes described in the present study occurred during cyclonic events that only indirectly impacted core-use areas. This indicates that the effects of hurricanes on coastal and marine taxa may extend well beyond those habitats centered on the eye of the storm. If changing global climate precipitates hurricanes of greater spatial extent [49], impacts to wildlife may be more widespread than previously reported.

## Conclusion

Hurricanes are acute meteorological disturbances that can act as significant environmental stressors to coastal and marine organisms. Despite the potential fitness consequences that they incur, species have adapted to the presence of episodic cyclonic events through behavioral modification and risk mitigation strategies. For Eastern brown pelicans in the South Atlantic Bight, this mitigation appears to be achieved through a decrease in movement and a prolonged maintenance of inactive behavior. These periods of rest occur in natural coastal structures such as barrier islands and estuarine systems, which provide shelter from many of the direct effects of hurricane exposure. While this strategy may be prevalent for large, coastal-dwelling seabirds, it is likely vastly different from strategies employed by other seabird guilds and by other marine vertebrate taxa, particularly those frequenting pelagic systems. Increased examination of animal movement responses to cyclonic events would greatly advance our understanding of how mobile organisms utilize behavioral modification to manage spatially-extensive environmental stressors, particularly in the face of climate change and the potential consequences for increased disruption therein.

## Data Availability

Data supporting this manuscript is available at www.sciencebase.gov, DOI: 10.5066/P9D5IP0G.

## Abbreviations

AIC: Akaike’s Information Criterion
EMbC: Expectation Maximization binary Clustering
G: Grams
HH: High velocity/ high turning angle
HL: High velocity/ low turning angle
Kmh: Kilometers per hour
KPa: Kilopascal
LH: Low velocity/ high turning angle
LL: Low velocity/ low turning angle
M: Meters
Min: Minutes
SD: Standard deviation
UD: Utilization distribution
